# Improving experimental reproducibility through comprehensive research protocols

**DOI:** 10.1002/2211-5463.70021

**Published:** 2025-04-01

**Authors:** Ivana Novak

**Affiliations:** ^1^ University of Split, School of Medicine Croatia

## Abstract

With this “In the Limelight: Research Protocols” special issue, *FEBS Open Bio* aims to highlight the critical importance of publishing reproducible and detailed scientific protocols that can be broadly adopted by laboratories working in molecular and cellular life sciences. This collection includes four protocols focused on sample preparation for structural analysis of macromolecules using X‐ray crystallography, covering both *in vitro* and *in vivo* approaches. Two additional protocols demonstrate the use of cellular systems to screen the enzymatic activity of various proteins, with potential applications in high‐throughput screening. Another protocol provides a comprehensive guide for the preparation and analysis of selective autophagy flux in cultured cells using flow cytometry. Finally, the issue concludes with a protocol integrating classical behavioral tests with cognitive components to assess both physical and cognitive dimensions of frailty.

In an era of rapidly advancing technologies that are significantly impacting science, it is increasingly clear that experimentation has become more challenging. In the field of molecular and cellular life sciences, it has always been crucial to obtain trustworthy, credible, and reproducible results. However, we are now also facing the rise of fast‐track publications, which often limit researchers' ability to provide detailed explanations of their experimental procedures, data acquisition, and analysis. To address this issue, *FEBS Open Bio* has curated this special issue, “In the Limelight: Research Protocols,” which features a collection of detailed experimental protocols in the molecular and cellular life sciences.

First, we present the three experimental approaches for the preparation of macromolecular crystals for crystallization since resolving the atomic structures of macromolecules is of constant interest to scientists for a precise understanding of the workings of cellular processes. Claude Sauter and his team provide details on the use of a new microfluidic chip developed in their laboratory, which enables the production of crystals via the counter‐diffusion method and their direct *in situ* structural analysis through serial crystallography at room temperature [1].

Kaščáková *et al*. [2] present a protocol for the production and crystallization of protein–ligand complexes, which is essential for studying detailed protein–ligand interactions, particularly beneficial in drug discovery. In a step‐by‐step manner, they highlight both cocrystallization and ligand soaking techniques. Additionally, they introduce an alternative approach for accelerating the crystallization process through microseeding.

The third crystallography protocol switches from *in vitro* to *in vivo* crystallization: Lars Radecke and his collaborators present a detailed experimental approach to the production of crystals inside living cells [3]. The group has recently published a streamlined pipeline for producing microcrystals within insect cells, and now they provide a comprehensive guide to improve the probability of obtaining intracellular crystals from proteins recombinantly produced in insect cells [4]. They additionally provide thoughtful insight on optimizing the results.

How to produce multiprotein complexes in insect cells using a baculovirus expression system for subsequent structural analysis by crystallography is in detail provided by Bitala *et al*. [5]. They emphasize how selenomethionine incorporation into native proteins early in the production process is beneficial for protein analysis. They describe an effective research protocol that combines protein labeling with simultaneous production of multiproteins using coinfection of cells with monocistronic baculoviruses. Furthermore, they provide a plethora of tips and tricks on how to help the implementation of this protocol in any laboratory.

Stuparević and collaborators describe a method for implementing a tool that utilizes genetically engineered yeast cells to produce cell wall proteins capable of anchoring any enzyme of interest to investigate their unknown functions [6]. This tool transforms yeast cell walls into a living catalytic material and could serve as a high‐throughput screening platform. The system outlined in this protocol allows for precise quantification and real‐time monitoring of enzymatic activity, providing a valuable and straightforward approach for studying enzyme kinetics and protein–ligand interactions. Furthermore, the research protocol by Živković *et al*. [7] also focuses on enzymatic activity analysis specifically targeting the enzymes that activate substrates at the expense of ATP. To address the limitations posed by the expensive and scarce [^32^P]‐PP_i_, the authors developed a method that replaces it with the more accessible [^32^P]‐γ‐ATP. Using aminoacyl‐tRNA synthetases as a model, the authors present an alternative that is affordable, reliable, robust, and extensively validated.

Work by Marinković *et al*. [8] presents the use of powerful flow cytometry to study fluorescently tagged receptors of selective autophagy. A detailed description of the preparation of cell samples for flow cytometry analysis is followed by an even more detailed postacquisition analysis. The authors emphasize that the protocol, although tested on mitophagy receptors, can be adapted to many other autophagy receptors with minor optimizations and used in laboratories with very simple flow cytometers.

Finally, Mladenović and Pracer developed a unique physical‐cognitive frailty score to measure frailty in rodents, which they bring in their research protocol [9]. Their aim is to equip researchers with improved tools for utilizing classical behavioral tests while incorporating a cognitive component to evaluate potential interventions targeting both the physical and cognitive dimensions of frailty.

I would like to express my gratitude to all the authors for their inspiring contributions to this special “In the Limelight” issue of *FEBS Open Bio*.

## Conflict of interest

The author declares no conflict of interest.

## Author contributions

IN wrote the editorial.

## References

[1] Pachl P , Coudray L , Vincent R , Nilles L , Scheer H , Ritzenthaler C , Fejfarová A , Řezáčová P , Engilberge S and Sauter C (2024) Protein crystallization and structure determination at room temperature in the crystal chip. FEBS Open Bio. doi: 10.1002/2211-5463.13932 PMC11961392 39572886

[2] Kaščáková B , Koutská A , Burdová M , Havlíčková P and Kutá Smatanová I (2024) Revealing protein structures: crystallization of protein‐ligand complexes–co‐crystallization and crystal soaking. FEBS Open Bio. doi: 10.1002/2211-5463.13913 PMC11961386 39428257

[3] Schönherr R , Eichler N , Sornaly FA , Boger J , Frevert AM , Lahey‐Rudolph JM , Meyer H , Weymar L and Redecke L (2025) Intracellular protein crystallization in living insect cells. FEBS Open Bio. 10.1002/2211-5463.70020 PMC11961387 40153432

[4] Schönherr R , Boger J , Lahey‐Rudolph JM , Harms M , Kaiser J , Nachtschatt S , Wobbe M , Duden R , König P , Bourenkov G *et al*. (2024) A streamlined approach to structure elucidation using in cellulo crystallized recombinant proteins, InCellCryst. Nat Commun 15, 1709. 38402242 10.1038/s41467-024-45985-7 PMC10894269

[5] Bitala A , Benko M , Nemčovič M and Nemčovičová I (2025) Equi‐MOI ratio for rapid baculovirus‐mediated multiprotein co‐expression in insect cells integrating selenomethionine for structural studies. FEBS Open Bio. 10.1002/2211-5463.70025 PMC11961385 40103323

[6] Martinić Cezar T , Paić A , Prekpalaj S , Teparić R , Žunar B and Stuparević I (2024) Measuring the capacity of yeast for surface display of cell wall‐anchored protein isoforms by using β‐lactamase as a reporter enzyme. FEBS Open Bio. doi: 10.1002/2211-5463.13886 PMC11961389 39198718

[7] Živković I , Dulic M , Kozulic P , Mocibob M and Gruic‐Sovulj I (2024) Kinetic characterization of amino acid activation by aminoacyl‐t RNA synthetases using radiolabelled γ‐[^32^P] ATP. FEBS Open Bio. doi: 10.1002/2211-5463.13903 PMC11961388 39344714

[8] Marinković M , Rožić A , Polančec D and Novak I (2024) Cost‐effective and simple flow cytometry quantification of receptor‐mediated autophagy using fluorescent tagging. FEBS Open Bio. doi: 10.1002/2211-5463.13958 PMC11961372 39716041

[9] Mladenović A and Pracer S (2024) Evaluating frailty scores across experimental groups in rodent models: bridging physical and cognitive domains. FEBS Open Bio. doi: 10.1002/2211-5463.13955 PMC11961374 39703035

